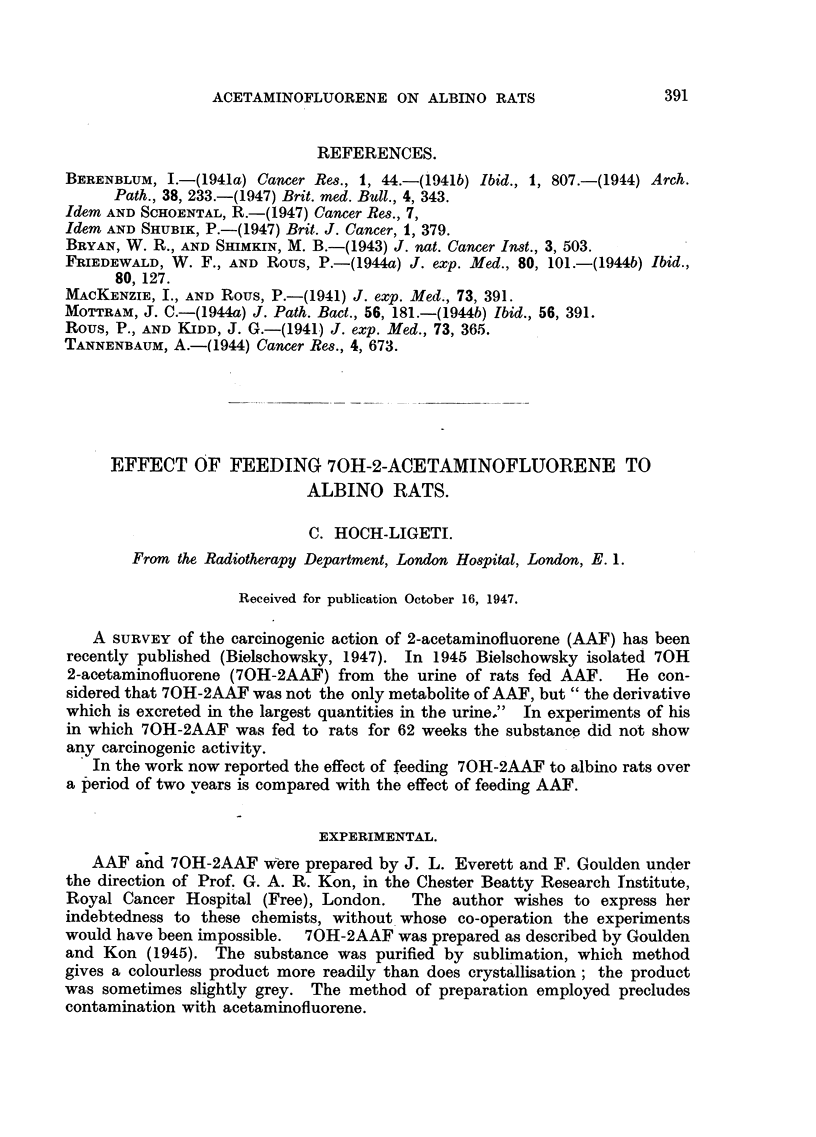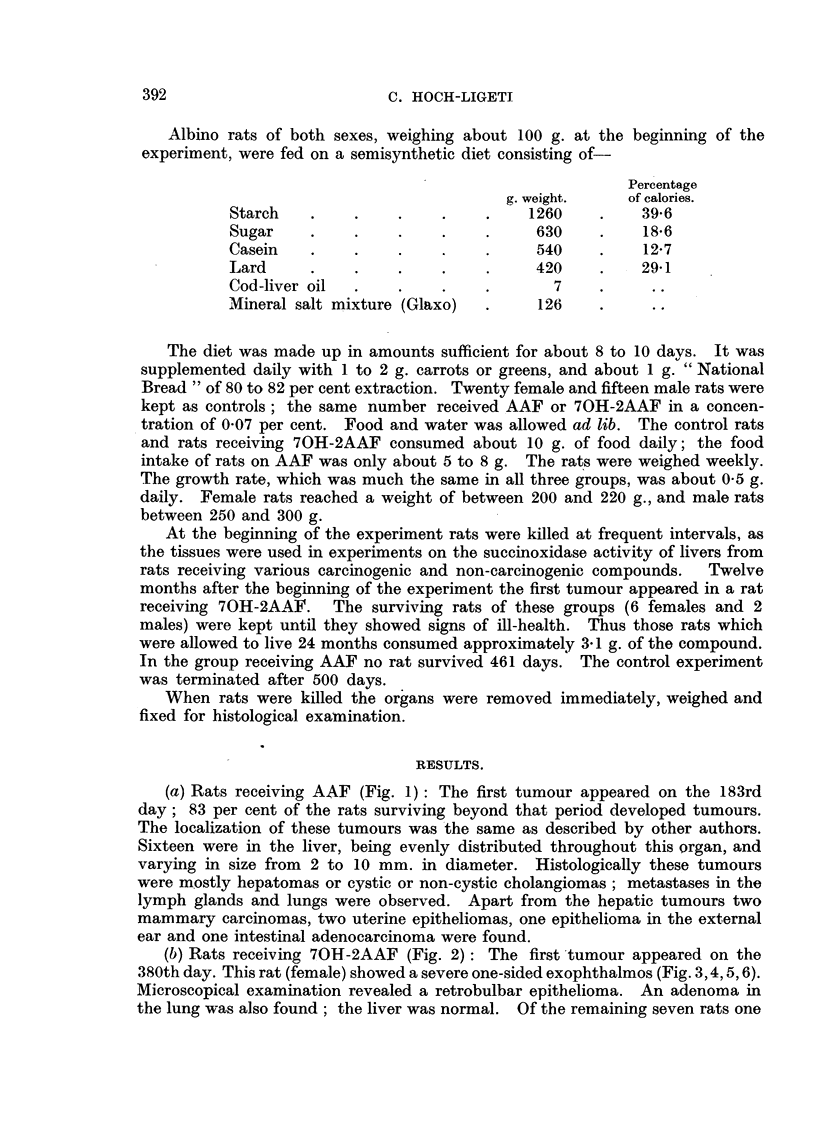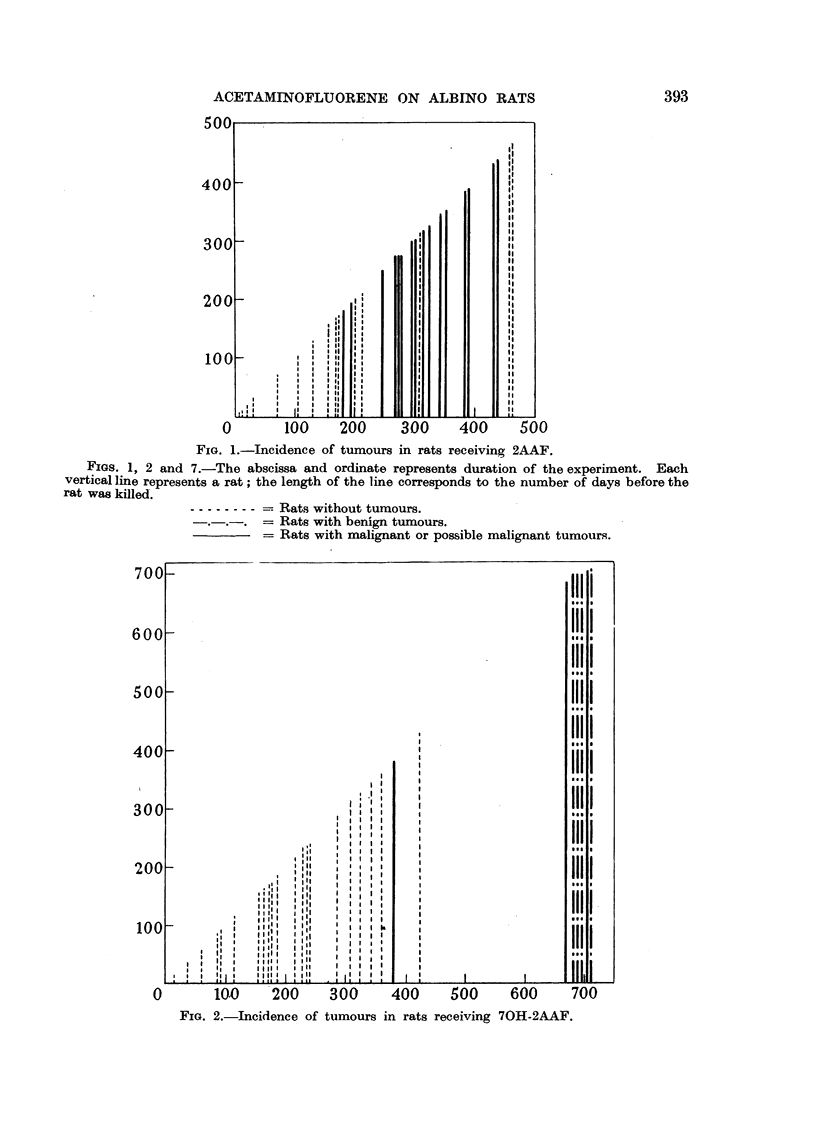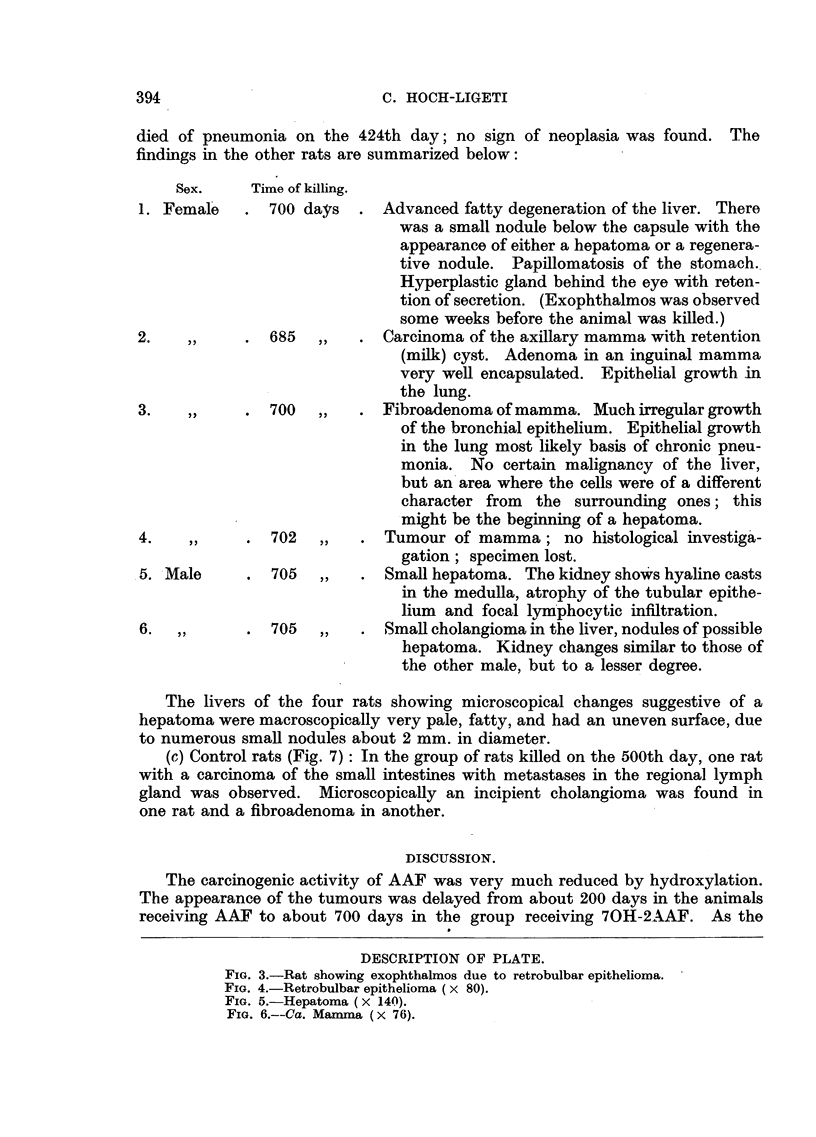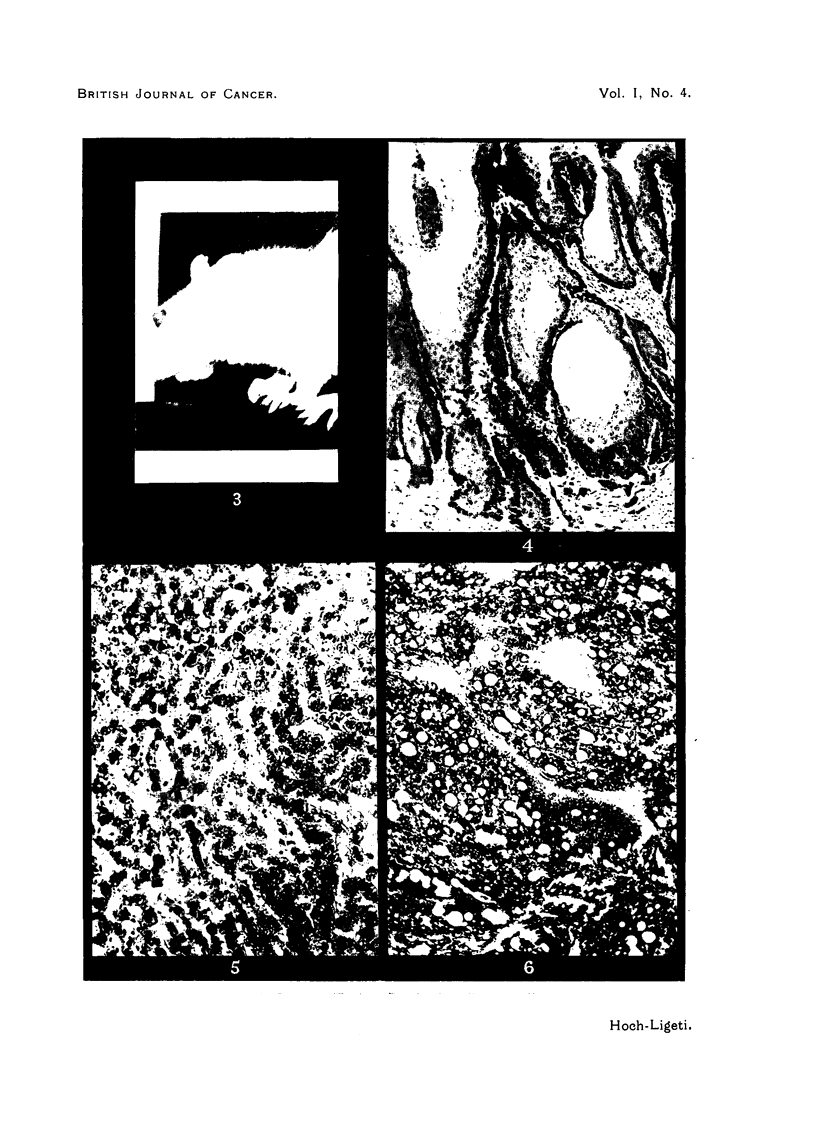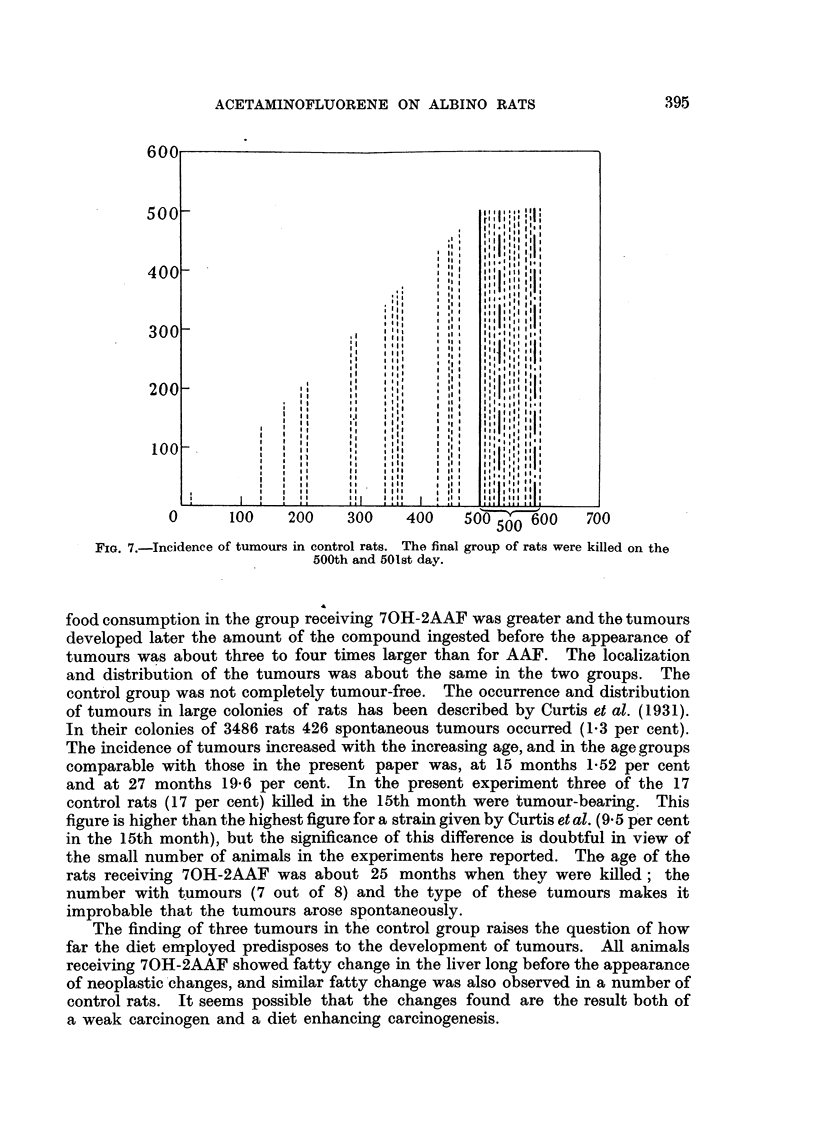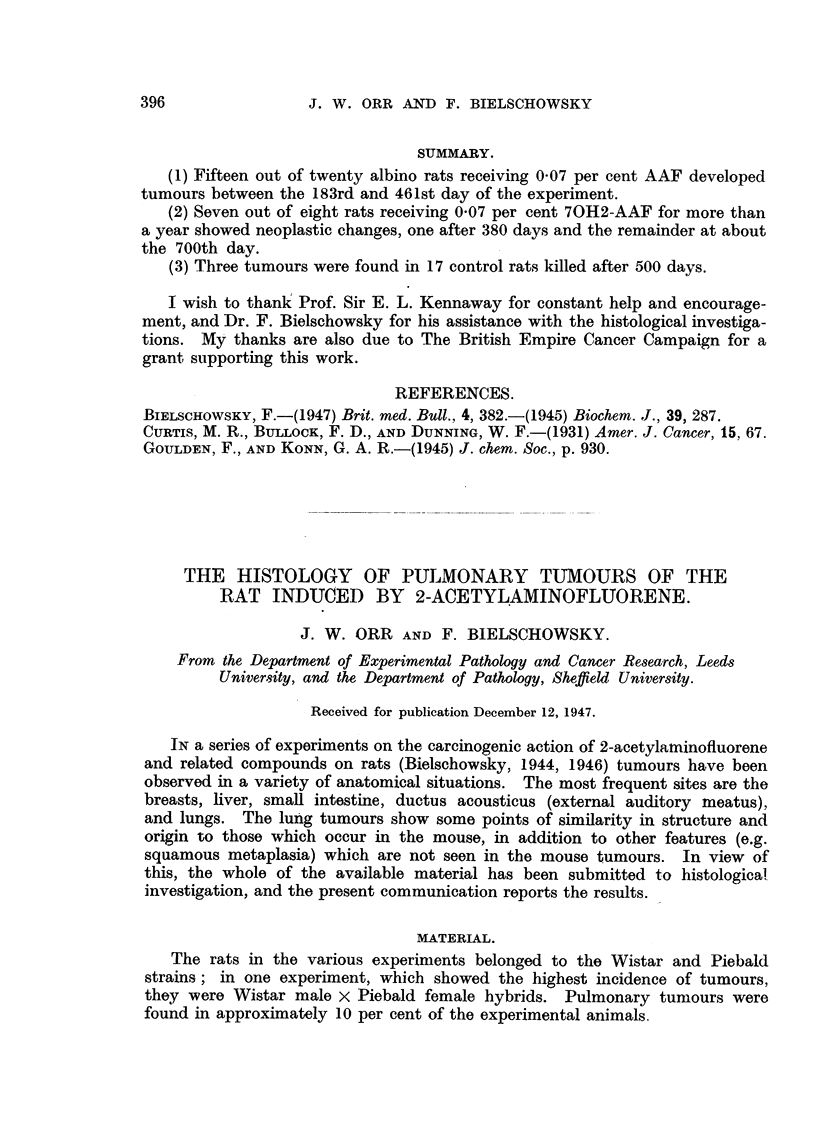# Effect of Feeding 7OH-2-Acetaminofluorene to Albino Rats

**DOI:** 10.1038/bjc.1947.37

**Published:** 1947-12

**Authors:** C. Hoch-Ligeti

## Abstract

**Images:**


REFERENCES.

### EFFECT OF FEEDING 7OH-2-ACETAMINOFLUORENE TO ALBINO RATS.

C. HOCH-LIGETI.

*From the Radiotherapy Department, London Hospital, London, E.* 1.

Received for publication October 16, 1947.

A SURVEY of the carcinogenic action of 2-acetaminofluorene (AAF) has been recently published (Bielschowsky, 1947). In 1945 Bielschowsky isolated 7OH 2-acetaminofluorene (7OH-2AAF) from the urine of rats fed AAF. He considered that 7OH-2AAF was not the only metabolite of AAF, but " the derivative which is excreted in the largest quantities in the urine." In experiments of his in which 7OH-2AAF was fed to rats for 62 weeks the substance did not show any carcinogenic activity.

In the work now reported the effect of feeding 7OH-2AAF to albino rats over a period of two years is compared with the effect of feeding AAF.

### EXPERIMENTAL.

AAF and 7OH-2AAF were prepared by J. L. Everett and F. Goulden under the direction of Prof. G. A. R. Kon, in the Chester Beatty Research Institute, Royal Cancer Hospital (Free), London. The author wishes to express her indebtedness to these chemists, without whose co-operation the experiments would have been impossible. 7OH-2AAF was prepared as described by Goulden and Kon (1945). The substance was purified by sublimation, which method gives a colourless product more readily than does crystallisation ; the product was sometimes slightly grey. The method of preparation employed precludes contamination with acetaminofluorene.

Albino rats of both sexes, weighing about 100 g. at the beginning of the experiment, were fed on a semisynthetic diet consisting of—

| | g. weight. | Percentage of calories. |
|---|---|---|
| Starch | 1260 | 39·6 |
| Sugar | 630 | 18·6 |
| Casein | 540 | 12·7 |
| Lard | 420 | 29·1 |
| Cod-liver oil | 7 | .. |
| Mineral salt mixture (Glaxo) | 126 | .. |

The diet was made up in amounts sufficient for about 8 to 10 days. It was supplemented daily with 1 to 2 g. carrots or greens, and about 1 g. "National Bread" of 80 to 82 per cent extraction. Twenty female and fifteen male rats were kept as controls; the same number received AAF or 7OH-2AAF in a concentration of 0·07 per cent. Food and water was allowed *ad lib.* The control rats and rats receiving 7OH-2AAF consumed about 10 g. of food daily; the food intake of rats on AAF was only about 5 to 8 g. The rats were weighed weekly. The growth rate, which was much the same in all three groups, was about 0·5 g. daily. Female rats reached a weight of between 200 and 220 g., and male rats between 250 and 300 g.

At the beginning of the experiment rats were killed at frequent intervals, as the tissues were used in experiments on the succinoxidase activity of livers from rats receiving various carcinogenic and non-carcinogenic compounds. Twelve months after the beginning of the experiment the first tumour appeared in a rat receiving 7OH-2AAF. The surviving rats of these groups (6 females and 2 males) were kept until they showed signs of ill-health. Thus those rats which were allowed to live 24 months consumed approximately 3·1 g. of the compound. In the group receiving AAF no rat survived 461 days. The control experiment was terminated after 500 days.

When rats were killed the organs were removed immediately, weighed and fixed for histological examination.

### RESULTS.

(*a*) Rats receiving AAF (Fig. 1): The first tumour appeared on the 183rd day; 83 per cent of the rats surviving beyond that period developed tumours. The localization of these tumours was the same as described by other authors. Sixteen were in the liver, being evenly distributed throughout this organ, and varying in size from 2 to 10 mm. in diameter. Histologically these tumours were mostly hepatomas or cystic or non-cystic cholangiomas; metastases in the lymph glands and lungs were observed. Apart from the hepatic tumours two mammary carcinomas, two uterine epitheliomas, one epithelioma in the external ear and one intestinal adenocarcinoma were found.

(*b*) Rats receiving 7OH-2AAF (Fig. 2): The first tumour appeared on the 380th day. This rat (female) showed a severe one-sided exophthalmos (Fig. 3, 4, 5, 6). Microscopical examination revealed a retrobulbar epithelioma. An adenoma in the lung was also found; the liver was normal. Of the remaining seven rats one

FIG. 1.—Incidence of tumours in rats receiving 2AAF.

FIGS. 1, 2 and 7.—The abscissa and ordinate represents duration of the experiment. Each vertical line represents a rat ; the length of the line corresponds to the number of days before the rat was killed.

- - - - - - - - = Rats without tumours.
—.—.—. = Rats with benign tumours.
———— = Rats with malignant or possible malignant tumours.

FIG. 2.—Incidence of tumours in rats receiving 7OH-2AAF.

died of pneumonia on the 424th day; no sign of neoplasia was found. The findings in the other rats are summarized below:

| | Sex. | Time of killing. | |
|---|---|---|---|
| 1. | Female | 700 days | Advanced fatty degeneration of the liver. There was a small nodule below the capsule with the appearance of either a hepatoma or a regenerative nodule. Papillomatosis of the stomach. Hyperplastic gland behind the eye with retention of secretion. (Exophthalmos was observed some weeks before the animal was killed.) |
| 2. | ,, | 685 ,, | Carcinoma of the axillary mamma with retention (milk) cyst. Adenoma in an inguinal mamma very well encapsulated. Epithelial growth in the lung. |
| 3. | ,, | 700 ,, | Fibroadenoma of mamma. Much irregular growth of the bronchial epithelium. Epithelial growth in the lung most likely basis of chronic pneumonia. No certain malignancy of the liver, but an area where the cells were of a different character from the surrounding ones; this might be the beginning of a hepatoma. |
| 4. | ,, | 702 ,, | Tumour of mamma; no histological investigation; specimen lost. |
| 5. | Male | 705 ,, | Small hepatoma. The kidney shows hyaline casts in the medulla, atrophy of the tubular epithelium and focal lymphocytic infiltration. |
| 6. | ,, | 705 ,, | Small cholangioma in the liver, nodules of possible hepatoma. Kidney changes similar to those of the other male, but to a lesser degree. |

The livers of the four rats showing microscopical changes suggestive of a hepatoma were macroscopically very pale, fatty, and had an uneven surface, due to numerous small nodules about 2 mm. in diameter.

(*c*) Control rats (Fig. 7): In the group of rats killed on the 500th day, one rat with a carcinoma of the small intestines with metastases in the regional lymph gland was observed. Microscopically an incipient cholangioma was found in one rat and a fibroadenoma in another.

### DISCUSSION.

The carcinogenic activity of AAF was very much reduced by hydroxylation. The appearance of the tumours was delayed from about 200 days in the animals receiving AAF to about 700 days in the group receiving 7OH-2AAF. As the

DESCRIPTION OF PLATE.

FIG. 3.—Rat showing exophthalmos due to retrobulbar epithelioma.
FIG. 4.—Retrobulbar epithelioma (× 80).
FIG. 5.—Hepatoma (× 140).
FIG. 6.—*Ca.* Mamma (× 76).

3

4

5

6

FIG. 7.—Incidence of tumours in control rats. The final group of rats were killed on the 500th and 501st day.

food consumption in the group receiving 7OH-2AAF was greater and the tumours developed later the amount of the compound ingested before the appearance of tumours was about three to four times larger than for AAF. The localization and distribution of the tumours was about the same in the two groups. The control group was not completely tumour-free. The occurrence and distribution of tumours in large colonies of rats has been described by Curtis *et al.* (1931). In their colonies of 3486 rats 426 spontaneous tumours occurred (1·3 per cent). The incidence of tumours increased with the increasing age, and in the age groups comparable with those in the present paper was, at 15 months 1·52 per cent and at 27 months 19·6 per cent. In the present experiment three of the 17 control rats (17 per cent) killed in the 15th month were tumour-bearing. This figure is higher than the highest figure for a strain given by Curtis *et al.* (9·5 per cent in the 15th month), but the significance of this difference is doubtful in view of the small number of animals in the experiments here reported. The age of the rats receiving 7OH-2AAF was about 25 months when they were killed ; the number with tumours (7 out of 8) and the type of these tumours makes it improbable that the tumours arose spontaneously.

The finding of three tumours in the control group raises the question of how far the diet employed predisposes to the development of tumours. All animals receiving 7OH-2AAF showed fatty change in the liver long before the appearance of neoplastic changes, and similar fatty change was also observed in a number of control rats. It seems possible that the changes found are the result both of a weak carcinogen and a diet enhancing carcinogenesis.

SUMMARY.

(1) Fifteen out of twenty albino rats receiving 0·07 per cent AAF developed tumours between the 183rd and 461st day of the experiment.

(2) Seven out of eight rats receiving 0·07 per cent 7OH2-AAF for more than a year showed neoplastic changes, one after 380 days and the remainder at about the 700th day.

(3) Three tumours were found in 17 control rats killed after 500 days.

I wish to thank Prof. Sir E. L. Kennaway for constant help and encouragement, and Dr. F. Bielschowsky for his assistance with the histological investigations. My thanks are also due to The British Empire Cancer Campaign for a grant supporting this work.

REFERENCES.

BIELSCHOWSKY, F.—(1947) *Brit. med. Bull.*, **4**, 382.—(1945) *Biochem. J.*, **39**, 287.
CURTIS, M. R., BULLOCK, F. D., AND DUNNING, W. F.—(1931) *Amer. J. Cancer*, **15**, 67.
GOULDEN, F., AND KONN, G. A. R.—(1945) *J. chem. Soc.*, p. 930.

---

### THE HISTOLOGY OF PULMONARY TUMOURS OF THE RAT INDUCED BY 2-ACETYLAMINOFLUORENE.

J. W. ORR AND F. BIELSCHOWSKY.